# Detection and Complete Genomic Analysis of *Porcine circovirus 3* (PCV3) in Diarrheic Pigs from the Dominican Republic: First Report on PCV3 from the Caribbean Region

**DOI:** 10.3390/pathogens12020250

**Published:** 2023-02-04

**Authors:** Kerry Gainor, Yussaira Castillo Fortuna, Angeline Steny Alakkaparambil, Wendy González, Yashpal Singh Malik, Souvik Ghosh

**Affiliations:** 1 Department of Biomedical Sciences, Ross University School of Veterinary Medicine, Basseterre P.O. Box 334, Saint Kitts and Nevis; 2 Faculty of Agronomic and Veterinary Sciences, Institute for the Study of Zoonotic Diseases, Autonomous University of Santo Domingo, Calle Camino de Engombe 10904, Dominican Republic; 3 Department of Biotechnology, School of Bio Sciences and Technology, Vellore Institute of Technology, Vellore 632014, India; 4 Epidemiological Surveillance Division, Dirección General de Ganadería 10410, Santo Domingo 10410, Dominican Republic; 5 School of Veterinary Medicine, Faculty of Agronomic and Veterinary Sciences, Autonomous University of Santo Domingo, Calle Camino de Engombe 10904, Dominican Republic; 6 College of Animal Biotechnology, Guru Angad Dev Veterinary and Animal Science University, Ludhiana 141012, India

**Keywords:** Caribbean region, complete genome analysis, diarrhea, Dominican Republic, pig, *Porcine circovirus 3*

## Abstract

The increasing detection of *Porcine circovirus 3* (PCV3, family *Circoviridae*) in clinically ill pigs worldwide has raised concerns on the implications of the virus on porcine health and the pork industry. Although pork production constitutes an important component of the livestock economy and is a major source of animal protein in the Caribbean Islands, there are no reports on PCV3 in pigs from the region so far. In the present study, PCV3 was detected in 21% (21/100) of diarrheic pigs (sampled at three farms) from the Caribbean nation of the Dominican Republic (DR). Although the sample size varied between porcine age groups, the highest PCV3 detection rates (35.3% each, respectively) were observed in piglets and growers. Co-infections with PCV2 and porcine adenovirus were observed in 38.09% and 9.52% of the PCV3 positive samples, respectively. The complete genomes of 11 DR PCV3 strains were analyzed in the present study, revealing a unique deletion (corresponding to nucleotide residue at position 1165 of reference PCV3 sequences) in one of the DR PCV3 sequences. Based on sequence identities and phylogenetic analysis (open reading frame 2 and complete genome sequences), the DR PCV3 strains were assigned to genotype PCV3a, and shared high sequence homologies (>98% identities) between themselves and with those of other PCV3a (Clade-1) strains, corroborating previous observations on the genetic stability of PCV3 worldwide. To our knowledge, this is the first report on the detection and molecular characterization of PCV3 in pigs from the Caribbean region, providing important insights into the expanding global distribution of the virus, even in isolated geographical regions (the Island of Hispaniola). Our findings warrant further investigations on the molecular epidemiology and economic implications of PCV3 in pigs with diarrhea and other clinical conditions across the Caribbean region.

## 1. Introduction

*Porcine circovirus 3* (PCV3), a member of the genus *Circovirus* (family *Circoviridae*), was first identified in diseased pigs from the United States in 2016, and since then has been increasingly detected in domestic pigs worldwide [1,2,3,4]. PCV3 has been reported in pigs with various clinical conditions (cardiac, gastrointestinal, integumentary, neurological, renal, reproductive, and respiratory disease, and multi-systemic inflammation) as well as in apparently healthy pigs [2,5,6,7]. Although experimental studies (in vitro/in vivo inoculation of virus, or infectious PCV3 DNA clones) have demonstrated evidence of PCV3 as a primary cause of certain clinical conditions, the pathogenesis of PCV3 remains to be clearly elucidated [2,6,7,8,9]. The increasing detection of PCV3 in co-infections with economically important porcine pathogens has further complicated the ongoing debate on the involvement of PCV3 in clinical disease, suggesting possible synergistic relationships between PCV3 and other porcine pathogens [2,5,6,7,8,9].

PCV3 has been found to be highly prevalent and endemic in wild boars, with evidence for long-lasting infection, suggesting their role as potential reservoirs and a source of the virus to domestic pigs [2,10,11,12,13]. Interestingly, PCV3 has also been detected in various non-*Suidae* species (cattle, chamois, dogs, donkeys, rodents, and roe deer), and in arthropods (mosquitoes and ticks), although the role/s of heterologous host/s or vectors in transmission of PCV3 remain to be properly interpreted [2,10,14,15,16,17].

*Porcine circovirus 3* shares a similar genomic organization with members of other viral species (*Porcine circovirus -1*, *-2,* and *-4* (PCV1, PCV2, and PCV4)) within the genus *Circovirus* [2,13]. The PCV3 genome consists of a circular, single-stranded ambisense DNA (~2000 bp) that contains at least three major open-reading frames (ORF): ORF1, ORF2, and ORF3, encoding a replication-associated (Rep) protein, a capsid (Cap) protein, and a protein with unknown function, respectively [1,2,5,13]. The PCV3 Cap plays an important role in virus attachment to host cells and is the primary target of the host immune system [2].

Since PCV3 strains share high sequence homologies (94.44–100% nucleotide (nt) homologies) between themselves, it has been challenging to establish a PCV3 genotype classification system [18,19]. Although different approaches have been attempted to genotype PCV3 strains, there were inconsistencies between the various classification schemes [2,18]. As a result, currently, there are no strict guidelines for the genotyping of PCV3 [2,13,18]. However, recently, Franzo et al. [18] defined a standardized criterion for genotyping PCV3 into 2 clades (Clade-1 and -2) based on genetic distance and phylogenetic analyses of the complete genome and ORF2 sequences.

In the Caribbean region, pork production represents a significant component of the livestock economy and is a major source of animal protein to the regional human population (~44 million) [20]. The Dominican Republic (located on the Caribbean Island of Hispaniola within the Greater Antilles (Supplementary Figure S1), human population of ~10 million) is one of the major pork producers (~1.8 million domestic pigs) in the Caribbean region (https://agricultura.gob.do, accessed on 18 October 2022). Several economically important porcine viral diseases (African swine fever, classical swine fever, porcine circovirus 2 (PCV2), porcine epidemic diarrhea (PED), porcine reproductive and respiratory syndrome (PRRS), porcine teschovirus-1, rabies, and swine influenza) have been reported in pigs from the Dominican Republic, although many of these reports were based on preliminary data [20]. Among the porcine circoviruses, to date, the molecular epidemiology of only PCV2 has been studied in the Dominican Republic [21] and in two other Caribbean islands (Cuba, and Saint Kitts and Nevis) [20,22]. On the other hand, although PCV3 has been increasingly detected in clinically ill pigs from different parts of the world, and the possible economic implications of PCV3 infections on pig farming are growing concerns, there are no reports on PCV3 in pigs from the Caribbean region so far [2,23]. We report here, for the first time, the detection and complete genome analysis of PCV3 in diarrheic pigs from the Caribbean nation of the Dominican Republic.

## 2. Materials and Methods

### 2.1. Sampling

The present study was based on archival fecal samples that were obtained from 100 diarrheic pigs for a previous research project on porcine enteric viruses (PCV2, PAdV, and RVA) in the Dominican Republic [21]. During August-November 2020, 34 and 16 fecal samples were obtained from diarrheic pigs at a farm in the municipality of Cabrera (housing ~300 pigs) and Pedro Brand (~100 pigs), respectively, and in January–February 2021, 50 fecal samples were collected from a pig farm in the municipality of Villa Mella (~500 pigs) (Supplementary Figure S1). The municipality of Cabrera is located on the northeastern coast of the Dominican Republic, whilst Pedro Brand and Villa Mella are located in the southeastern part of the country.

When the diarrheic pig defecated, the attending veterinarian held a sterile container (4 oz. Specimen Cup, Dynarex Corporation, New York, NY, USA) near the rectal orifice of the animal and collected a sample of the liquid/semi-liquid feces. Since all pigs were tagged on the farms, we could avoid sampling the same animal twice. After the sample was obtained, the container was sealed with sterile tape, packed in two layers of biohazard bags, and transferred under cold chain to the laboratory. To prevent contamination, gloves and personal protective equipment were changed between collection of samples. The samples were stored at −20 °C until laboratory analysis.

The sampled animals belonged to different porcine age groups: weaners (aged >3 to ≤10 weeks) (35%, 35/100), growers (>10 to ≤18 weeks) (17%, 17/100), piglets (0 to 3 weeks) (17%, 17/100), farrow/pregnant (13%, 13/100), boar (3%, 3/100), gilt (1%, 1/100), and dry sows (14%, 14/100). During sampling, most of the growers and weaners experienced retarded growth, pallor, and reduced body weight. Although we could not obtain specific information on porcine reproductive health in the three pig farms, abortions, small litter size, metritis, and repetition of estrous were important concerns in Cabrera, Pedro Brand, and Villa Mella. Previously, animals on the farm in Pedro B ≤rand had tested positive for *Mycoplasma* and PRRSV, with several weaners exhibiting clinical signs of respiratory distress. On the other hand, the farm in Cabrera had tested positive for *Actinobacillus pleuropneumonia* and PRRSV, whilst the farm in Villa Mella had reported PRRSV. However, we could not obtain specific information on the numbers of animals infected with these pathogens on the farms. Official approval by the Institutional Animal Care and Use Committee (IACUC) of the Ross University School of Veterinary Medicine (RUSVM), Saint Kitts and Nevis, was granted for the collection and use of the porcine fecal samples for the present study (RUSVM IACUC #: TSU6.10.22).

### 2.2. Amplification of PCV3 Genome

Extraction of viral DNA from the fecal samples was carried out using the QIAamp Fast DNA Stool Mini Kit (Qiagen Sciences, Germantown, MD, USA) based on the manufacturer’s instructions. The porcine fecal samples were screened for the presence of PCV3 DNA by a PCV3-specific nested PCR assay targeting the ORF2, as previously described [24]. Briefly, a 649 bp region (nt 1339-nt 1987 of strain PCV3/CN/Fujian-5/2016) of the PCV3 ORF2 was amplified using primers PCV3-1-F (5′-TTACTTAGAGAACGGACTTGTAACG-3′) and PCV3-1-R (5′-AAATGAGACACAGAGCTATATTCAG-3′), followed by a nested PCR using internal primers 5′-CCATTGAACGGTGGGGTCAT-3′ (nt 1442–nt 1461) and 5′- TGGACCACAAACACTTGGCT-3′ (nt 1645-nt 1626) [24]. The PCR conditions were: initial denaturation at 95 °C for 3 min, followed by 35 cycles of 95 °C for 30 s, 54 °C for 1 min, 72 °C for 1 min (30 s for the second PCR), and a final extension at 72 °C for 7 min. Amplification of the complete genomes of PCV3 strains were achieved using three overlapping nested PCRs designed in the present study (Supplementary Table S1). All PCRs were carried out using the Platinum™ Taq DNA Polymerase (Invitrogen™, Thermo Fisher Scientific Corporation, Waltham, MA, USA), following the manufacturers’ recommendations. The porcine fecal samples were also screened for the presence of porcine deltacoronavirus (PDCoV), PEDV, and transmissible gastroenteritis virus (TGEV) using broad spectrum primers targeting the coronavirus RNA-dependent RNA polymerase gene in a pan-RT-PCR assay as described in a previous study [25]. The QIAGEN One-Step RT-PCR Kit (Qiagen Sciences, Germantown, MD, USA) was used for performing the RT-PCR reactions following the manufacturers’ instructions. Sterile water was used as the negative control in all PCR assays.

### 2.3. Nucleotide Sequencing

Purification of the PCR products was carried out using the Wizard^®^ SV Gel and PCR Clean-Up kit (Promega, Madison, WI, USA) according to the instructions of the manufacturer. Nucleotide sequences were determined using the ABI Prism Big Dye Terminator Cycle Sequencing Ready Reaction Kit on an ABI 3730XL Genetic Analyzer (Applied Biosystems, Foster City, CA, USA).

### 2.4. Sequence Analysis

Homology searches for related nt and deduced amino acid (aa) sequences and pairwise sequence identities (using the ‘align two or more sequences’ option) were determined using the standard BLASTN and BLASTP programs (Basic Local Alignment Search Tool, www.ncbi.nlm.nih.gov/blast, accessed on 18 October 2022), respectively. The ORF finder (https://www.ncbi.nlm.nih.gov/orffinder/, accessed on 18 October 2022) was employed to determine the putative ORFs. Multiple alignments of deduced aa and nt sequences were performed using the Clustal Omega (https://www.ebi.ac.uk/Tools/msa/clustalo, accessed on 18 October 2022) and CLUSTALW (https://www.genome.jp/tools-bin/clustalw, accessed on 18 October 2022) programs, respectively.

The complete genomes of PCV3 strains were investigated for potential recombination events by the RDP4 program with default parameters and were recognized as recombinants if supported by 2 or >2 detection methods (3Seq, CHIMAERA, BOOTSCAN, MAXCHI, GENECONV, RDP, and SISCAN) with a highest acceptable *p*-value of *p* < 0.01 after Bonferroni’s correction, as previously described [21,26]. Phylogenetic analyses were performed by the MEGA7 software using the maximum-likelihood method with the Hasegawa–Kishino–Yano (HKY) model plus Gamma (G) distribution, and supported with 1000 bootstrap replicates, as described previously [7,18,23,27]. Genetic distances (p-distance) were calculated using the MEGA7 software [27].

### 2.5. GenBank Accession Numbers

The GenBank accession numbers for the complete genome sequences of the PCV3 strains from the Dominican Republic are OP616720-OP616730.

## 3. Results and Discussion

In the present study, 26 out of 100 fecal samples from diarrheic pigs in the Dominican Republic yielded the expected ~200 bp amplicon with the PCV3-specific nested PCR assay targeting a partial stretch of the PCV3 ORF2 [24]. All the 26 PCR products were sequenced to confirm the presence of PCV3 DNA. Based on BLASTN analysis, 21 of the 26 sequences shared maximum homology with PCV3 strains. On the other hand, the remaining 5 sequences were found to share high identities with non-PCV3 genomes, suggesting false positive PCR reactions. Therefore, 21% (21/100) of the diarrheic pigs were positive for PCV3 in the present study.

The rates of detection of PCV3 on the pig farm in Villa Mella, Cabrera, and Pedro brand were 14/50 (28%), 7/34 (20.6%), and 0/16 (0%), respectively (Table 1). Although PCV3 has been reported in all stages of pig production, some researchers have proposed that the prevalence of PCV3 is highest in piglets and declines with age, whilst other studies did not find any correlation between PCV3 detection rates and different porcine age groups [5,9,28,29,30,31,32]. In the present study, the rates of detection of PCV3 were higher in piglets (6/17; 35.3%) and growers (6/17; 35.3%) compared to other porcine age groups that were represented with a sufficient number of samples (weaners (7/35; 20%), dry sows (1/14; 7.1%), and farrow/pregnant sows (0/13, 0%)), whilst those in boars and gilts were 33.3% (1/3) and 0% (0/1), respectively (Table 1). Since the sample size varied between age groups, we could not determine the age predilection of PCV3.

PCV3 co-infections with important porcine pathogens are frequently encountered in pig populations worldwide [2,5,6,7,9,31,32]. It has been speculated that PCV3 might function synergistically with other porcine pathogens to potentiate disease severity [5,9,31,32]. On the other hand, co-infections might be due to the widespread occurrence of other major porcine pathogens, such as PCV2, devoid of any association with PCV3 [9,28,33]. In previous studies, we screened the 100 porcine fecal samples from the Dominican Republic for PCV2 (detection rates of 48%, 48/100 fecal samples) and porcine adenovirus (PAdV) (26%, 26/100 fecal samples) DNA, and *Rotavirus-A* (RVA) RNA (0%, 0/100 fecal samples) [21,34]. In the present study, the fecal samples were also screened for the presence of porcine coronaviruses (PDCoV, PEDV, and TGEV) using a pan-coronavirus RT-PCR assay as described previously [25]. PCV2 and PAdV were detected in eight (38.09%) and two (9.52%) of the PCV3 positive samples, respectively (Table 1). On the other hand, none of the tested fecal samples were positive for porcine coronaviruses or RVA. Other studies have also reported high detection rates of PCV3 in conjunction with PCV2 from diseased pigs [2,31,32,33]. Taken together, these observations warrant further investigation on the possible role/s of PCV3 in porcine co-infections, especially with PCV2.

Although PCV3 positivity rates of 10.4–27.28% have been reported in diarrheic pigs under field conditions (including the present study), experimental inoculation studies with PCV3 have revealed conflicting results regarding the induction of diarrhea and pathological changes in the small intestine, and there are conflicting reports on PCV3 detection rates between diseased and healthy pigs [2,8,31,35,36,37,38,39]. Since the present study was based on archival fecal samples from pigs with diarrhea, we could not compare the PCV3 detection rates between diarrheic and healthy/other clinically ill animals. As a result, our findings might not reflect the actual PCV3 situation on the pig farms. Furthermore, the PCV3 positive fecal samples were screened for only a few porcine pathogens (PAdV, PCV2, porcine coronaviruses, and, RVA), revealing high rates of co-infections with PCV2, and therefore, we could not establish the role of PCV3 in porcine diarrhea.

A total of 11 PCV3 positive fecal samples from the Dominican Republic (DR) (representing two of the pig farms that reported PCV3, different sampling periods and production stages) were available in sufficient volumes required for characterization of the whole PCV3 genome. The complete genomes of the DR PCV3 strains were of the same size (2000 bp in length) as those of most other PCV3 strains [23,40], except for a single DR strain (strain Po/PCV3/DOM/P4/2021, 1999 bp in size). Strain P4 exhibited a unique deletion corresponding to ‘G’ residue at position 1165 (located between ORF1 and 2) of reference PCV3 strain PCV3/CN/Fujian-5/2016 (GenBank accession number KY075986). Similar deletion events have been reported at nearby positions (corresponding to ‘G’ residue at position 1155 [41] and position 1148 [42], respectively) within the PCV3 genome, although their implications are not known. All the DR PCV3 sequences retained the various features (putative origin of replication (*ori*) marked by a nonanucleotide motif (TAGTATTAC) and certain motifs in the putative Rep) that are conserved among members of the genus *Circovirus* [1,4,35] (Supplementary Figure S2).

The DR PCV3 strains shared ORF1, ORF2, and complete genome nt sequence identities of 99.33–100%, 99.53–100%, and 99.55–100%, respectively, between themselves. With other PCV3 strains, the DR strains shared high ORF1 and ORF2 nt sequence identities of 99.33–99.89% and 99.38–99.69% with PCV3 strains from China (GenBank accession numbers MF677839, MF769808, and KY865243) and Italy (MF805722, MF805721, and MF805719), respectively, whilst high complete genome nt sequence identities of 99.40–99.65% were observed with PCV3 strains from Brazil (MK645718) and Spain (MW167067). Since recombination events have been shown to influence circovirus evolution, with possible implications on virulence and antigenicity [43], the complete genome sequences of the DR PCV3 strains were screened for potential recombinant viruses. However, the RDP4 program [26] did not reveal any evidence for recombination events involving the DR PCV3 strains (data not shown).

The putative Rep (296 aa) and Cap (214 aa) of the DR PCV3 strains shared deduced aa sequence identities of 98.31–100% and 100% between themselves, and maximum identities of 98.65–100% and 100% with those of other PCV3 strains, respectively. Since the circovirus Cap has been associated with viral virulence and is responsible for inducing host immune responses [1,2,13,40,44], the putative Cap of the DR PCV3 strains were evaluated for aa substitutions. Multiple alignment of the putative Cap sequences revealed 4 aa mismatches (A24V, R27K, T77S, and L150I) between the DR PCV3 strains and PCV3 reference strain NC_031753 (Supplementary Figure S3). Although these aa mismatches have been reported in several other PCV3 strains, limited information is available on the consequences of mutations in PCV3 genomes [23,40]. It has been proposed that A24V and R27K might be associated with immune escape, whilst aa residue at position 77 and 150 has been predicted to be in the outermost region of the viral capsid and believed to be under positive selection [23,40,45,46,47,48].

**Figure 1:**
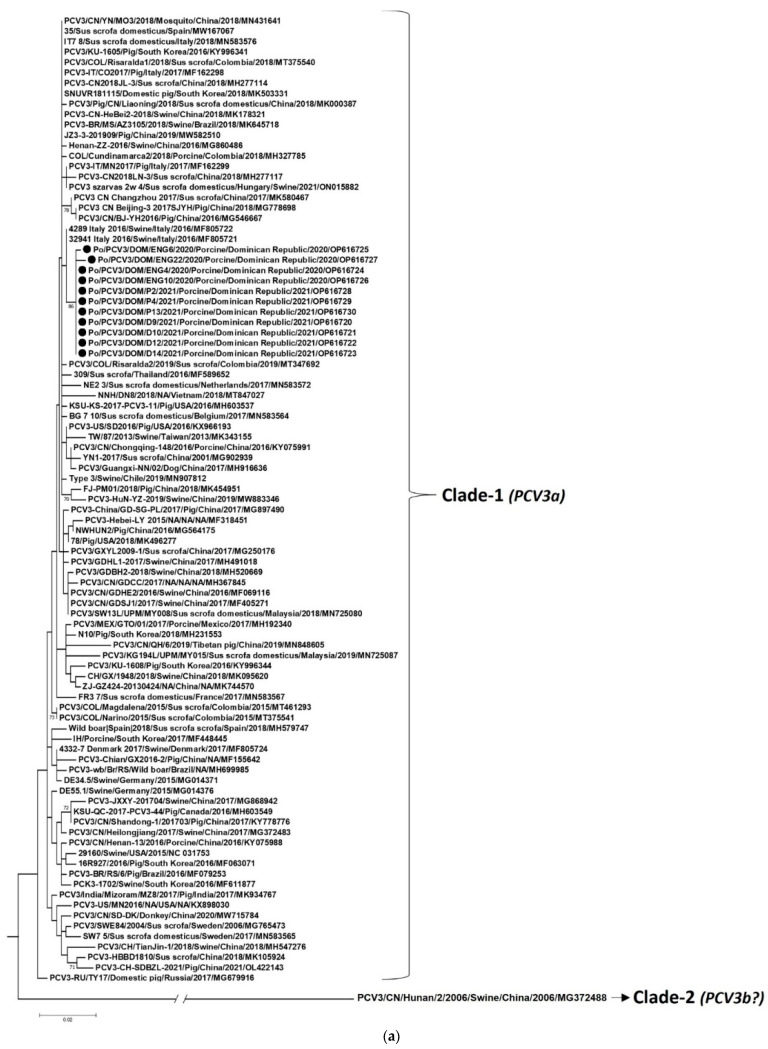

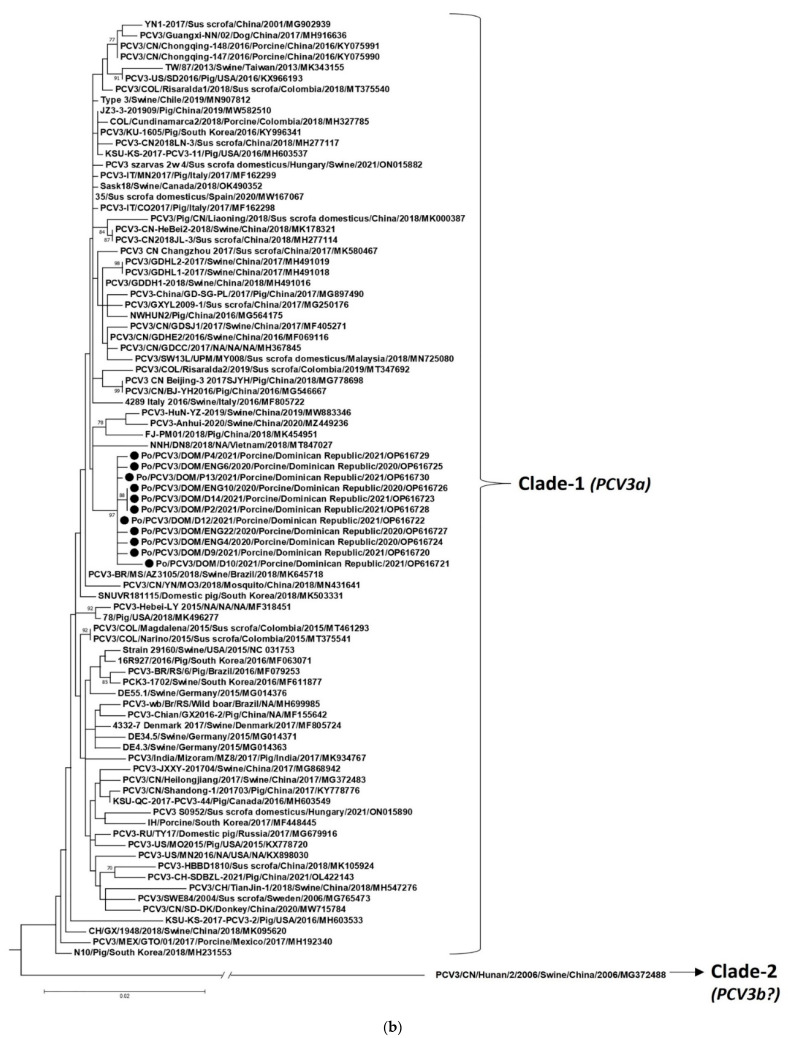
Phylogenetic analyses of the nucleotide sequences of open reading frame 2 (**a**) and complete genomes (**b**) of *Porcine circovirus 3* (PCV3) strains from the Dominican Republic with those of other PCV3 strains representing different countries. The strain name/year/host species/country/year/GenBank accession number are shown for all PCV3 strains. Black circles indicate the PCV3 strains detected from the Dominican Republic. The classification scheme by Franzo et al. [18] does not formally recognize PCV3 strains within Clade-2 as a genotype. Although Clade-2 consisted of two PCV3 sequences, GenBank accession number MG372490 was removed at the submitter’s request (https://www.ncbi.nlm.nih.gov/nuccore/MG372490, accessed on 18 October 2022), and could not be included in the present analysis. In figure (**a**,**b**), porcine circovirus 2 strain 22625-33-PCV2/Swine/USA/2012/JX535296 was used as the outgroup sequence (not shown here due to space constraints). Bootstrap values <65% are not shown. Scale bar, 0.02 substitutions per nucleotide.

In the present study, the classification scheme proposed by Franzo et al. [18] was employed to genotype the DR PCV3 strains. Based on a bootstrap support of 90%, maximum within-genotype genetic distance of 3% and 6% at the complete genome and ORF2 levels, respectively, and concordance in findings between ORF2 and complete genome analysis, Franzo et al. [18] classified PCV3 strains into two clades: Clade-1 and -2. Almost all PCV3 strains from different countries belonged to Clade-1 (p-distance range: 0.000–0.026 (complete genome) and 0.000–0.024 (ORF2)) and were designated as genotype PCV3a, whilst Clade-2 consisted of only 2 PCV3 sequences from China [18]. Further sub-classification of PCV3 was not suggested due to the lack of a meaningful association between PCV3 and its biological/epidemiological features [18]. Phylogenetically, the full-length ORF2 (p-distance range: 0.000–0.029) and complete genome (0.000–0.018) sequences of the DR PCV3 strains exhibited similar clustering patterns, forming a tight cluster within the PCV3 Clade-1 in respective trees (Figure 1a,b), and based on the recommendations of Franzo et al. [18], were assigned to genotype PCV3a.

To our knowledge, this is the first report on the detection and molecular characterization of PCV3 in pigs from the Caribbean region, providing important insights into the increasing global distribution of the virus, even in isolated geographical regions (the Island of Hispaniola). It might be possible that PCV3 was introduced into the Dominican Republic through the importation of animals and/or animal products from nearby North and/or South American countries where the virus has already been reported in porcine populations [23]. Analysis of the ORF2 and complete genome sequences assigned all DR PCV3 strains to genotype PCV3a, corroborating previous observations on the genetic stability (low mutation rates) of PCV3 worldwide [2,18,23]. Although the present study had limitations: (i) was not based on a massive sample size (archival fecal samples from 100 diarrheic pigs in three farms); (ii) apparently healthy animals were not tested in the present study; and (iii) fecal samples were screened by a nested PCR assay, whilst qPCR was used in several other studies [9], the detection of PCV3 in 21% (21/100) of diarrheic pigs warrants further investigation on the molecular epidemiology and economic implications of PCV3 in pigs with enteritis and other clinical conditions across the Caribbean region.

## Figures and Tables

**Table 1:** Details of the diarrheic pigs that tested positive for *Porcine circovirus 3* (PCV3) in the Dominican Republic.

Animal/Sample Number	Age Group/Category of Animal ^1^	Location of the Farm	Year of Sample Collection	*Porcine circovirus 2*	*Porcine adenovirus*
ENG4	Growers/fatteners	Cabrera ^2^	2020	+	+
ENG6	Growers/fatteners	Cabrera	2020	+	−
ENG9	Growers/fatteners	Cabrera	2020	+	−
ENG10	Growers/fatteners	Cabrera	2020	+	−
ENG11	Growers/fatteners	Cabrera	2020	+	−
ENG22	Growers/fatteners	Cabrera	2020	+	−
VE22	Boar	Cabrera	2020	+	+
PP14	Dry Sow	Villa Mella ^2^	2021	−	−
P1	Piglet	Villa Mella	2021	−	−
P2	Piglet	Villa Mella	2021	−	−
P4	Piglet	Villa Mella	2021	+	−
P5	Piglet	Villa Mella	2021	−	−
P11	Piglet	Villa Mella	2021	−	−
P13	Piglet	Villa Mella	2021	−	−
D7	Weaner	Villa Mella	2021	−	−
D8	Weaner	Villa Mella	2021	−	−
D9	Weaner	Villa Mella	2021	−	−
D10	Weaner	Villa Mella	2021	−	−
D12	Weaner	Villa Mella	2021	−	−
D14	Weaner	Villa Mella	2021	−	−
D21	Weaner	Villa Mella	2021	−	−

**^1^** Based on porcine age groups/animal categories defined by the National Farm Animal Care Council (NFACC), Canada (https://www.nfacc.ca/codes-of-practice/pig-code#glossary, accessed on 18 October 2022); **^2^** Municipality in the Dominican Republic.

## Data Availability

Additional data are available Supplementary Table S1 and Figures S1–S3.

## Supplementary Materials

The following supporting information can be downloaded at: https://www.mdpi.com/article/10.3390/pathogens12020250/s1, Supplementary Table S1: Primers used in three overlapping nested PCR assays to amplify the complete genomes of Porcine circovirus 3 (PCV3) strains from the Dominican Republic. Supplementary Figure S1. Map of the Dominican Republic showing the porcine sampling sites. Supplementary Figure S2. The conserved rolling circle replication motifs (I-yellow; II-red; III-dark blue), superfamily 3 helicase motifs (Walker A-green; B-purple; C-orange) and motifs of unknown function (I-brown; II-black; III-sky blue) were retained in the putative replication-associated proteins (Rep) of the Porcine circovirus 3 (PCV3) strains (strain name/year/host species/country/year/GenBank accession number) from the Dominican Republic. Numbers to the right indicate amino acid positions for respective strains. Supplementary Figure S3. Multiple alignment of the deduced amino acid (aa) sequences of the putative capsid (Cap) proteins of the 11 Porcine circovirus 3 (PCV3) strains from the Dominican Republic (DR) (strain name/year/host species/country/year/GenBank accession number) with the Cap of PCV3 reference strain (strain name/year/host species/country/year/GenBank accession number) NC_031753 (www.ncbi.nlm.nih.gov/genbank/, accessed on 15 October 2022). The Cap of DR PCV3 strains exhibited 4 aa mismatches (position 24, 27, 77 and 150, shown with green, yellow, red, and blue box, respectively) with that of strain NC_031753. Numbers to the right indicate aa position for respective strains.
