# Supplementary material for: Detection and Complete Genomic Analysis of Porcine circovirus 3 (PCV3) in Diarrheic Pigs from the Dominican Republic: First Report on PCV3 from the Caribbean Region
Source: Pathogens. 2023 Feb 4;12(2):250. doi: 10.3390/pathogens12020250 (PMC9959359; doi:10.3390/pathogens12020250)
Supplement: Supplementary file 1 [file pathogens-12-00250-s001.zip › 1_Supplementary Figure S1.pptx]

## Slide 1
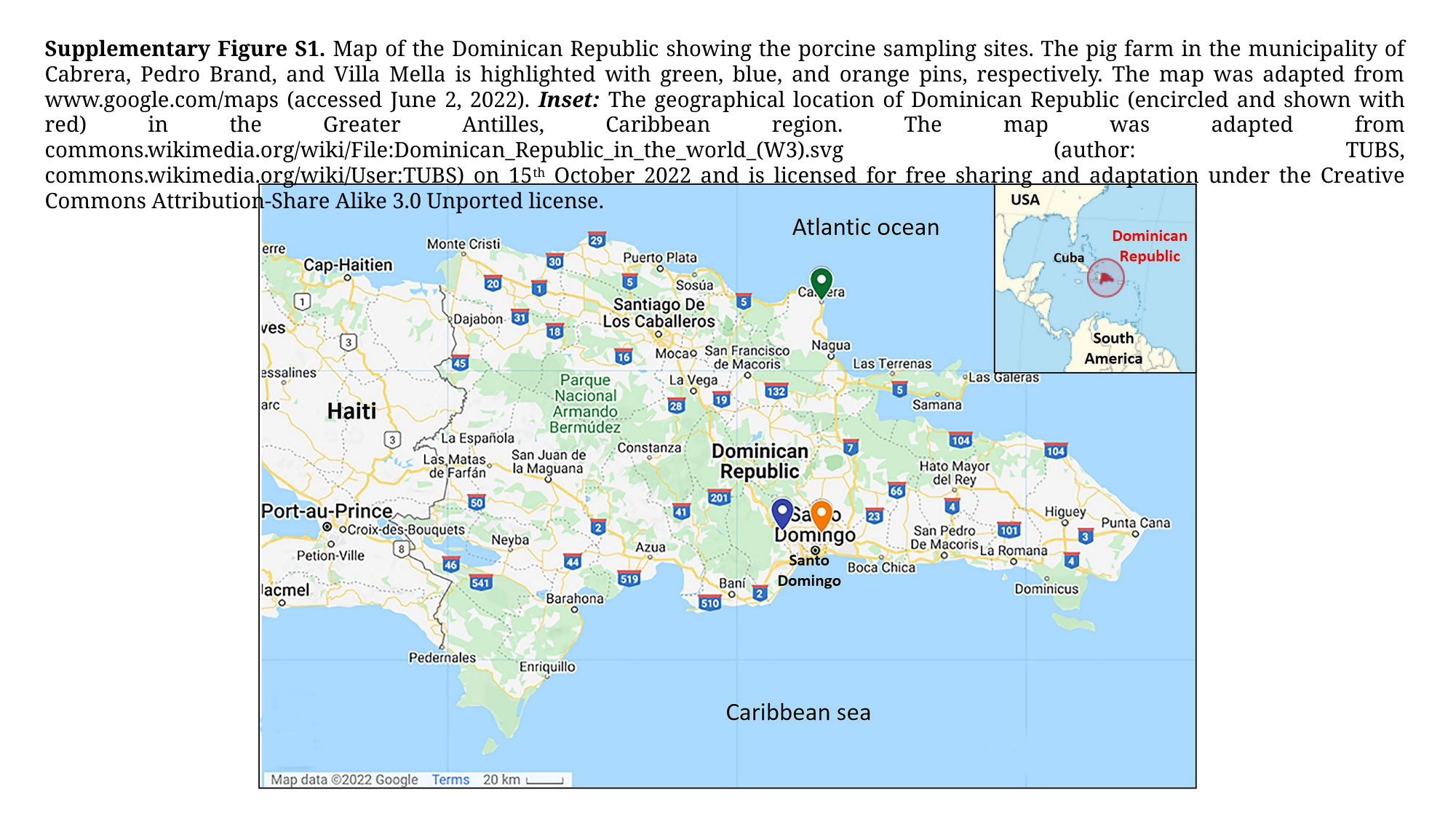

Supplementary Figure S1. Map of the Dominican Republic showing the porcine sampling sites. The pig farm in the municipality of Cabrera, Pedro Brand, and Villa Mella is highlighted with green, blue, and orange pins, respectively. The map was adapted from www.google.com/maps (accessed June 2, 2022). Inset: The geographical location of Dominican Republic (encircled and shown with red) in the Greater Antilles, Caribbean region. The map was adapted from commons.wikimedia.org/wiki/File:Dominican_Republic_in_the_world_(W3).svg (author: TUBS, commons.wikimedia.org/wiki/User:TUBS) on 15th October 2022 and is licensed for free sharing and adaptation under the Creative Commons Attribution-Share Alike 3.0 Unported license.
