# Supplementary material for: Detection and Complete Genomic Analysis of Porcine circovirus 3 (PCV3) in Diarrheic Pigs from the Dominican Republic: First Report on PCV3 from the Caribbean Region
Source: Pathogens. 2023 Feb 4;12(2):250. doi: 10.3390/pathogens12020250 (PMC9959359; doi:10.3390/pathogens12020250)
Supplement: Supplementary file 1 [file pathogens-12-00250-s001.zip › 2_Supplementary Figure S2.pptx]

## Slide 1
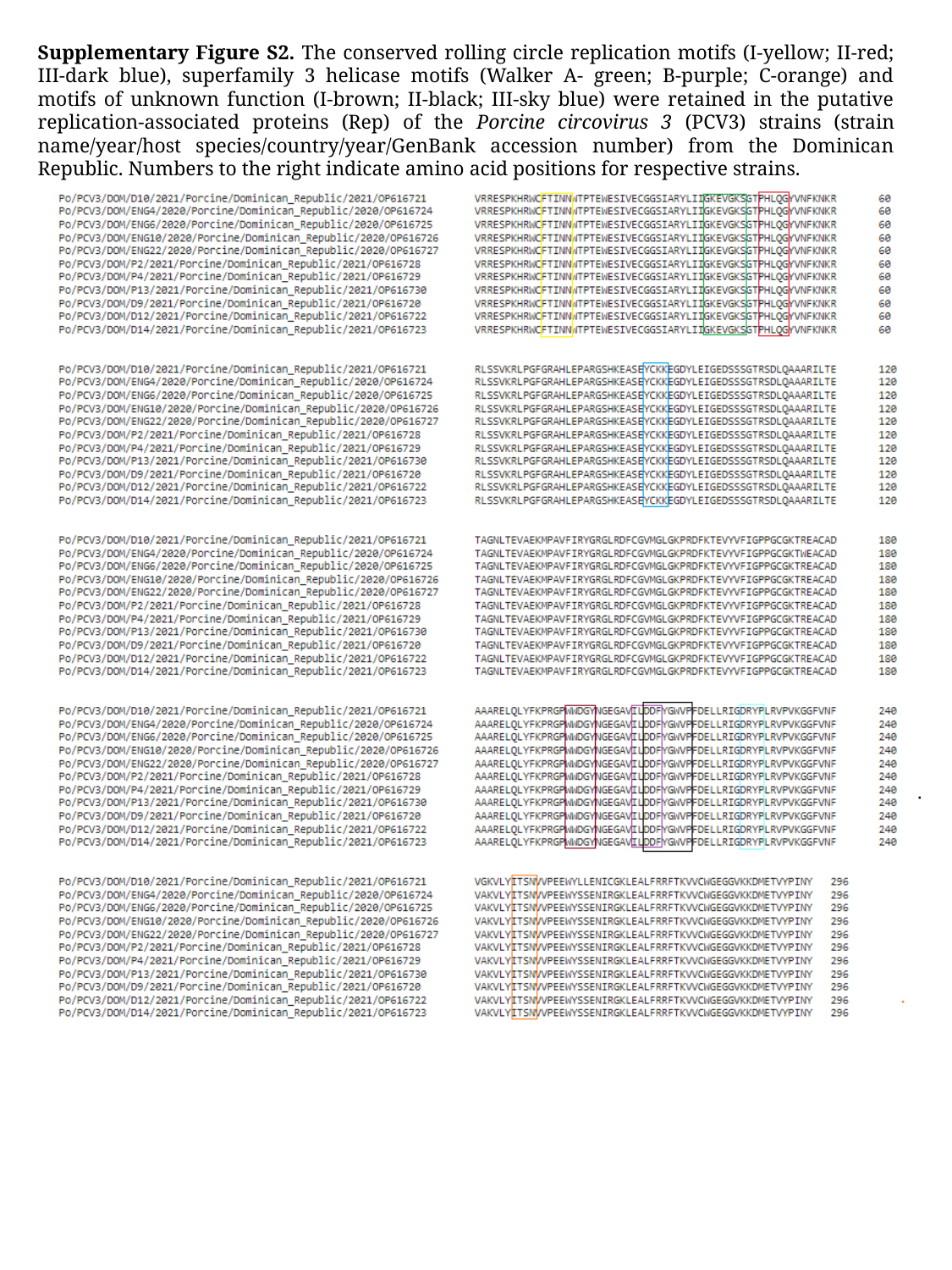

Supplementary Figure S2. The conserved rolling circle replication motifs (I-yellow; II-red; III-dark blue), superfamily 3 helicase motifs (Walker A- green; B-purple; C-orange) and motifs of unknown function (I-brown; II-black; III-sky blue) were retained in the putative replication-associated proteins (Rep) of the Porcine circovirus 3 (PCV3) strains (strain name/year/host species/country/year/GenBank accession number) from the Dominican Republic. Numbers to the right indicate amino acid positions for respective strains.
