# Supplementary material for: Detection and Complete Genomic Analysis of Porcine circovirus 3 (PCV3) in Diarrheic Pigs from the Dominican Republic: First Report on PCV3 from the Caribbean Region
Source: Pathogens. 2023 Feb 4;12(2):250. doi: 10.3390/pathogens12020250 (PMC9959359; doi:10.3390/pathogens12020250)
Supplement: Supplementary file 1 [file pathogens-12-00250-s001.zip › 3_Supplementary Figure S3.pptx]

## Slide 1
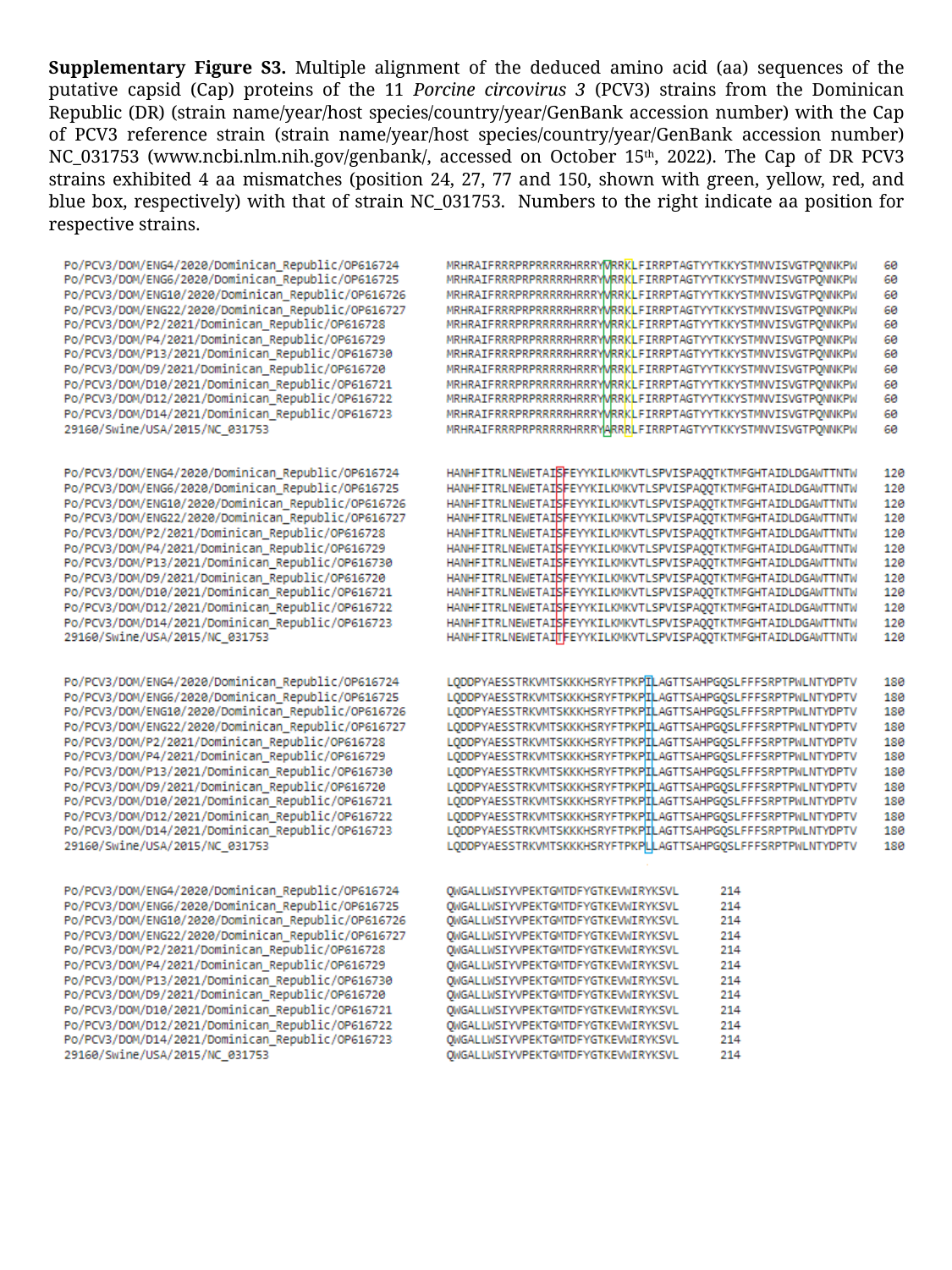

Supplementary Figure S3. Multiple alignment of the deduced amino acid (aa) sequences of the putative capsid (Cap) proteins of the 11 Porcine circovirus 3 (PCV3) strains from the Dominican Republic (DR) (strain name/year/host species/country/year/GenBank accession number) with the Cap of PCV3 reference strain (strain name/year/host species/country/year/GenBank accession number) NC_031753 (www.ncbi.nlm.nih.gov/genbank/, accessed on October 15th, 2022). The Cap of DR PCV3 strains exhibited 4 aa mismatches (position 24, 27, 77 and 150, shown with green, yellow, red, and blue box, respectively) with that of strain NC_031753. Numbers to the right indicate aa position for respective strains.
