# Supplementary material for: Detection and Complete Genomic Analysis of Porcine circovirus 3 (PCV3) in Diarrheic Pigs from the Dominican Republic: First Report on PCV3 from the Caribbean Region
Source: Pathogens. 2023 Feb 4;12(2):250. doi: 10.3390/pathogens12020250 (PMC9959359; doi:10.3390/pathogens12020250)
Supplement: Supplementary file 1 [file pathogens-12-00250-s001.zip › 4_Supplementary Table S1.pdf]

Supplementary Table S1. Primers used in three overlapping nested PCR assays to amplify the complete genomes of *Porcine circovirus 3* (PCV3) strains from the Dominican Republic.

**Nested PCR reaction**		**Primer name**	**Primer sequence † (5′-3′)**	**Primer position ‡**
**A**	First PCR	PCV3-F-A<br>PCV3-R-A	TGAGTGGATATACCGGGCAGTG<br>TCCGATGAATACCACTCCTCCG	nt 88-nt 109<br>nt 1011-nt 990
	Second PCR	PCV3-FN-A<br>PCV3-RN-A	GTGTTTTGATGCCGCAGGAC<br>CCACAAAATTAACAAACCCACCC	nt 127-nt 146<br>nt 946-nt 924
**B**	First PCR	PCV3-F-B<br>PCV3-R-B	GGGTTTGCGTGATTTTTGCGG<br>AAACACAGCCGTTACTTCACCC	nt 645-nt 665<br>nt 1570-nt 1549
	Second PCR	PCV3-FN-B<br>PCV3-RN-B	GATGGGGTTGGGTAAACCGC<br>TTCTGGCGGGAACTACCAGC	nt 669-nt 688<br>nt 1539-nt 1520
**C**	First PCR	PCV3-F-C<br>PCV3-R-C	ATGCGAGGGCGTTTACCTGTG<br>CTTCACCGAGCTGAGTCGCC	nt 1238-nt 1258<br>nt 420-nt 401
	Second PCR	PCV3-FN-C<br>PCV3-RN-C	CCACCGGAGGGGTCAGATTT<br>GTACCCTTGCAAGTGTGGGG	nt 1309-nt 1328<br>nt 381-nt 362

† The primer sequences were determined from conserved regions among PCV3 genomes.

‡ Nucleotide (nt) positions are those of reference PCV3 strain PCV3/CN/Fujian-5/2016 (GenBank accession number KY075986).
